# Supplementary material for: Systematic mapping of altermagnetic magnons by resonant inelastic X-ray circular dichroism
Source: Nat Commun. 2025 Oct 21;16:9311. doi: 10.1038/s41467-025-64322-0 (PMC12541013; doi:10.1038/s41467-025-64322-0)
Supplement: Supplementary file 1 — Supplementary Information [file 41467_2025_64322_MOESM1_ESM.pdf]

Supplementary Information for "Systematic Mapping of Altermagnetic Magnons by Resonant Inelastic X-Ray Circular Dichroism"

Nikolaos Biniskos,[1,*] Manuel dos Santos Dias,[2,†] Stefano Agrestini,[3]
David Sviták,[1] Ke-Jin Zhou,[3] Jiří Pospíšil,[1] and Petr Čermák[1]

[1]*Charles University, Faculty of Mathematics and Physics, Department of Condensed Matter Physics, Ke Karlovu 5, 121 16, Praha, Czech Republic*

[2]*Scientific Computing Department, STFC Daresbury Laboratory, Warrington WA4 4AD, United Kingdom*

[3]*Diamond Light Source, Harwell Campus, Didcot, OX11 0DE, United Kingdom*

(Dated: September 8, 2025)

CONTENTS

I. Supplementary experimental information 2
A. Synthesis and characterization of CrSb 2
B. X-ray absorption spectra, resonant inelastic X-ray scattering measurements and data analysis 2

II. Supplementary theoretical information 4
A. DFT 4
B. Symmetry and the magnetic exchange interactions 5
C. Altermagnetic magnons 8
D. RIXS and dichroism of the magnon peak 10

Supplementary References 14

* nikolaos.biniskos@matfyz.cuni.cz
† manuel.dos-santos-dias@stfc.ac.uk

I. SUPPLEMENTARY EXPERIMENTAL INFORMATION

A. Synthesis and characterization of CrSb

High quality single crystals of CrSb have been grown directly from the stoichiometric amount of pure elements (Cr: 4N, Sb: 6N) using the chemical vapour transport (CVT) method. We used iodine as a transport agent in the ratio 50 mg to ~ 3 g of input material. The ampule was evacuated down to vacuum of 10^{-6} mbar before sealing. A thermal gradient of $850/750^{\circ}$ C was kept for three weeks during the single crystal growth and approximately 1/3 of the starting material still remained untransported. Plate-like single crystals of silver metallic reflective colour with regular hexagon shapes were obtained, and a few exceeded a cm-scale. The desired 1:1 composition was confirmed by energy dispersive X-ray (EDX) analysis. In addition, a small amount of single crystals was crushed to powder and X-ray powder diffraction (XRPD) was performed to confirm the quality and single phase of the samples. The crystallinity and orientation of the single crystals were confirmed by the Laue method at room temperature showing sharp reflections. The hexagonal c-axis is perpendicular to the plane of the plate-like crystals. A Néel temperature of $T_{\mathrm{N}} = 685$ K was determined by differential scanning calorimetry (DSC) performed in the temperature range $300 \leq T \leq 1000$ K.

B. X-ray absorption spectra, resonant inelastic X-ray scattering measurements and data analysis

Resonant inelastic X-ray scattering (RIXS) experiments were performed at the I21 beamline of the Diamond Light Source in the UK [1]. CrSb single crystals were cleaved prior to the beam time, aligned in the [100]/[001] scattering plane of the hexagonal symmetry and mounted on a copper sample holder using silver based epoxy. The pressure in the experimental chamber was maintained below $5 \cdot 10^{-10}$ mbar. The used photon energy was around the Cr L_3 edge. X-ray absorption spectroscopy (XAS) obtained at $T = 300$ K before the RIXS measurements is based on the total fluorescence yield method. RIXS data were obtained at $T = 300$ K using circular right (CR) and circular left (CL) polarizations of the incident X-ray beam. The energy resolution is estimated as $\Delta E = 32.5$ meV from the full-width of the half-maximum (FWHM) of the elastic peak from an adjacent carbon tape. Every RIXS

spectrum is normalised to the incident flux, measured via the focusing mirror current, which was collected simultaneously with the RIXS spectrum with the same acquisition time. In short, the RIXS intensity is divided by the M4 mirror current.

The zero-energy transfer position was first roughly determined by the elastic peak position of a carbon tape that was mounted next to the sample and was finely adjusted during the fitting procedure of the RIXS spectra. The fitting model of the RIXS spectra consists of a Voigt function for the elastic peak, a Gaussian function for the magnon peak and a background that fits the tail of the fluorescence signal at higher energy.

We anticipate the existence of large and homogeneous AFM domains in CrSb without external magnetic field, by comparison with the results obtained for another uniaxial collinear AFM, Cr_2O_3 [2], and given the small footprint of the beam ($\sim 30\,\mu\text{m} \times 2\,\mu\text{m}$) this will allow the measurements of RIXS MCD in single magnetic domains (see Fig. S1). Using the dichroism of the RIXS magnon peak as a proxy for the orientation of the Néel vector, a scan across the sample allowed us to determine that the domain sizes in CrSb are of the order of several hundreds of micrometres, i.e. much larger than the small footprint of the beam. For the azimuthal scans two positions of the domains A and B were selected well away from the domain border and separated by $500\,\mu\text{m}$ (see Fig. S1). A cross overlap to the image of the optical micro-camera provides a visual view of the location of the beam on the sample. The beam position on the sample was recorded and checked using the optical camera after each azimuthal rotation. There was a small drift in the sample position after each rotation but given the large domain separation and the small beam size, all the RIXS MCD were confidently measured well within each single magnetic domain (A or B). The RIXS spectra for the azimuthal dependence ϕ of the spin-wave excitations were obtained in the same θ_f angle, while keeping the scattering angle fixed. Therefore, self-absorption corrections that depend mainly on the experimental geometry and energy loss of the photons at a given incident photon energy were not applied to the spectra.

XAS spectra were collected at two incidence angles θ_i, 20.5° and 90.5°. In altermagnets analogous to FM the XAS-XMCD signal is expected to maximize when the helicity vector of the photon is parallel (CR) or anti-parallel (CL) to the sample magnetization, therefore, a decrease is expected with decreasing θ_i from normal ($\theta_i = 90°$) to grazing ($\theta_i = 0°$) incidence. Although the XMCD spectra were obtained well below the Néel temperature, they exhibit no difference at the two incidence angles and consequently no magnetically

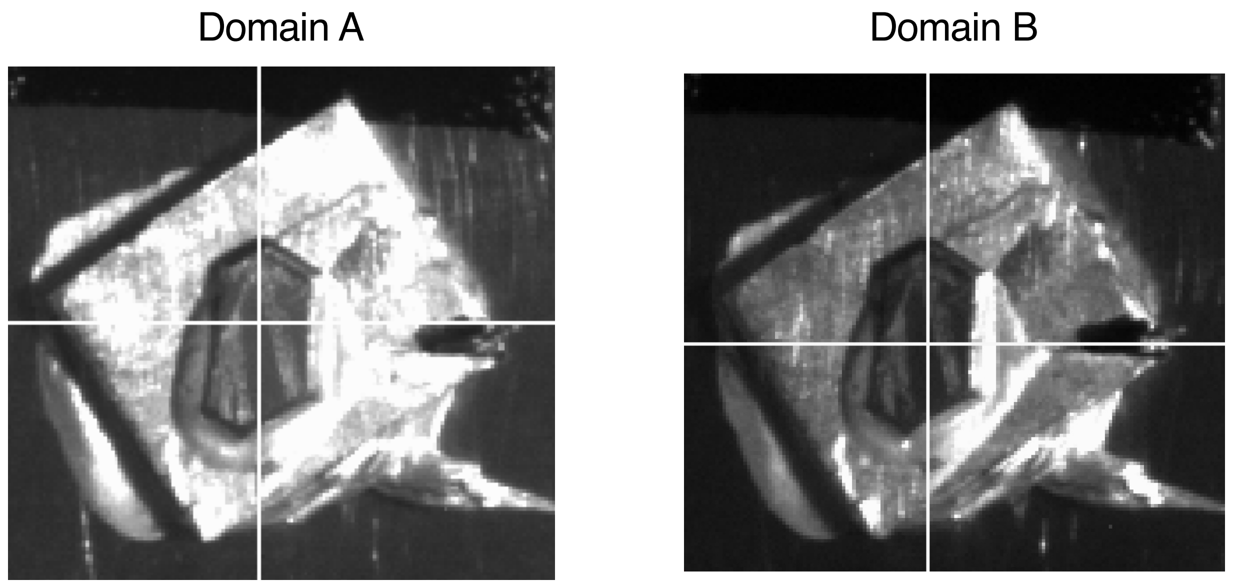

Fig. S1. **Sample positions of two different antiferromagnetic domains.** The center of the cross indicates the position where RIXS spectra were collected at $\phi = 0°$ in domain A and B. The distance between the two measurement spots in the vertical direction is 500 μm.

dichroic phenomena. The spectra taken at normal incidence are shown in Fig. 2a of the main text; measurements taken with a $\theta_i = 20.5°$ incidence angle are shown in Fig. S2(a). An overview of the RIXS spectra for various photon incident energies is shown in Fig. S2(b), with the energy loss region where the magnon peak is visible in Fig. S2(c).

II. SUPPLEMENTARY THEORETICAL INFORMATION

A. DFT

We theoretically explore the magnetic properties of CrSb using DFT with the the juKKR code package [3], which implements the all-electron Korringa-Kohn-Rostoker Green's function method using full potential [4] and spin-orbit coupling added to the scalar relativistic approximation [5]. The exchange-correlation functional is the local spin-density approximation as parameterized by Vosko, Wilk and Nusair [6]. The scattering wave functions are expanded up to an angular momentum cutoff of $\ell_{\max} = 3$, the energy integrations are carried out in the upper complex energy plane with a Fermi-Dirac smearing of $T = 502.78\,\mathrm{K}$ [7], and the Brillouin zone summations use a k-point grid of $16 \times 16 \times 12$ points. The juKKR

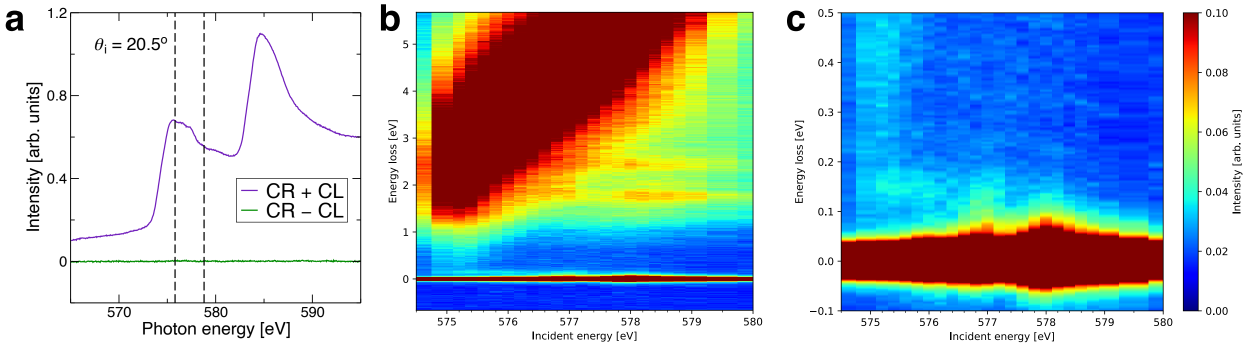

Fig. S2. **Complementary measurements.** **a** X-ray absorption and dichroic spectra from total fluorescence yield measurements using incident X-rays with circular-right (CR) and circular left (CL) polarization with an incidence angle of $\theta_i = 20.5°$. The vertical dashed lines indicate the selected photon energies for RIXS. **b** RIXS spectra over a wide energy loss range, showing the dominant fluorescence continuum and sharp *d*-*d* excitations. **c** Zoomed-in view of **b** showing the magnon signal around 0.15 eV.

code package gives access to the tensor of pairwise magnetic exchange interactions via the relativistic extension of the infinitesimal rotation approach [8], which we use to parameterize the spin Hamiltonian given in Eq. S1 below.

The Cr magnetic moment is computed to be $2.78\,\mu_\mathrm{B}$, and Fig. S3 displays the spin-resolved local density of states for the *d*-orbitals of the Cr atom at the unit cell origin. The Fermi energy is almost at the edge of the minority *d*-states, which explains why the computed exchange interactions are very sensitive to its precise placement.

B. Symmetry and the magnetic exchange interactions

Consider an antiferromagnetic crystal with the magnetic sites located at $\mathbf{R}_{i\mu} = \mathbf{R}_i + \mathbf{R}_\mu$, with $\mathbf{R}_i$ the sites of a Bravais lattice and $\mathbf{R}_\mu$ the basis vectors for the two sublattices in the reference unit cell, $\mathbf{R}_i = 0$, with sublattice labels $\mu = \mathrm{A}, \mathrm{B}$. Let the spin Hamiltonian be

$$\mathcal{H} = -\frac{1}{NS^2} \sum_{i,j} \sum_{\mu,\nu} J_{ij}^{\mu\nu}\, \mathbf{S}_i^\mu \cdot \mathbf{S}_j^\nu \,. \tag{S1}$$

Here N is the number of sites in the Bravais lattice and S is the magnitude of the spins; $S = 3/2$ is consistent with both the theoretical and experimental value of the spin magnetic moment. The $1/S^2$ prefactor makes the $J_{ij}^{\mu\nu}$ parameters independent of the choice of S, and

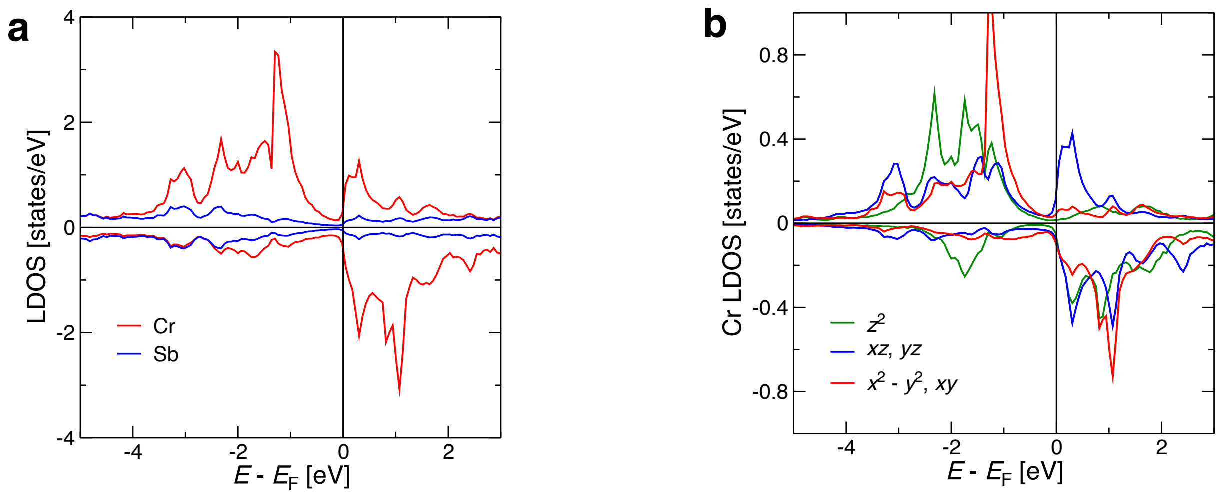

Fig. S3. **Electronic structure of CrSb.** **a** Local density of states projected on Cr and on Sb. **b** Local density of states projected on Cr resolved into d-orbital components.

so we can use the values computed with DFT directly in this form. With this sign convention, J_{ij}^{AA} and J_{ij}^{BB} should be predominantly positive to stabilise ferromagnetism within each sublattice, and J_{ij}^{AB} should be predominantly negative to stabilise the antiferromagnetic coupling between the two sublattices.

The lattice Fourier transform of the exchange interactions is defined to be:

$$J^{\mu\nu}(\mathbf{k}) = \frac{1}{N}\sum_{i,j} J_{ij}^{\mu\nu}\, e^{\mathrm{i}\mathbf{k}\cdot(\mathbf{R}_j-\mathbf{R}_i)}\, e^{\mathrm{i}\mathbf{k}\cdot(\mathbf{R}_\nu-\mathbf{R}_\mu)} = \sum_{j} J_{0j}^{\mu\nu}\, e^{\mathrm{i}\mathbf{k}\cdot\mathbf{R}_{0j}^{\mu\nu}}\ , \tag{S2}$$

where $\mathbf{R}_{0j}^{\mu\nu} = \mathbf{R}_j + \mathbf{R}_\nu - \mathbf{R}_\mu$ This is independent of i by translational invariance of the exchange interactions and is invariant under the symmetries of the crystal. The additional phase factor $e^{\mathrm{i}\mathbf{k}\cdot(\mathbf{R}_\nu-\mathbf{R}_\mu)}$ simplifies substantially the derivations. We have four types of couplings: $J^{\mathrm{AA}}(\mathbf{k})$, $J^{\mathrm{BB}}(\mathbf{k})$, $J^{\mathrm{AB}}(\mathbf{k})$ and $J^{\mathrm{BA}}(\mathbf{k})$. These are constrained by the point group symmetry operations $\mathcal{O}$:

$$\mathcal{O}J^{\mu\nu}(\mathbf{k})\mathcal{O}^{-1} = \sum_{j}\mathcal{O}J_{0j}^{\mu\nu}\mathcal{O}^{-1}\, e^{\mathrm{i}\mathbf{k}\cdot\mathbf{R}_{0j}^{\mu\nu}} = \sum_{j'} J_{0j'}^{\mu'\nu'}\, e^{\mathrm{i}\mathbf{k}\cdot\left(\mathcal{O}^{-1}\mathbf{R}_{0j'}^{\mu'\nu'}\right)} = J^{\mu'\nu'}(\mathcal{O}^{-1}\mathbf{k})\ , \tag{S3}$$

using $\mathbf{R}_{0j'}^{\mu'\nu'} = \mathcal{O}\mathbf{R}_{0j}^{\mu\nu}$.

In the high-temperature paramagnetic phase, CrSb has the space group $P6_3/mmc$ (No. 194) which is taken to be the symmetry of the exchange interactions. The Cr atoms are in the Wyckoff position $2a$ with point group symmetry $\bar{3}m$ and the Sb atoms are in the Wyckoff position $2c$ with point group symmetry $\bar{6}m2$. Sublattice A is defined by Cr at $(0, 0, 0)$ and

sublattice B by Cr at $(0, 0, {}^1\!/_2)$, with the Sb at $({}^2\!/_3, {}^1\!/_3, {}^1\!/_4)$ and $({}^1\!/_3, {}^2\!/_3, {}^3\!/_4)$. One can show that $J^{\mathrm{AA}}(\mathbf{k})$ and $J^{\mathrm{AB}}(\mathbf{k})$ are invariant under the $\bar{3}m$ and $\bar{6}m2$ symmetries, which include inversion, and so these lattice Fourier transforms are real: $J^{\mathrm{AA}}(\mathbf{k})^* = J^{\mathrm{AA}}(-\mathbf{k}) = J^{\mathrm{AA}}(\mathbf{k})$. The simplest way to relate BB to AA is through a mirror plane m_z that passes through the origin and is perpendicular to the c-axis. This swaps the positions of the Sb atoms in the unit cell, so that now the environment of the Cr atom at the origin is the same as for Cr B in the actual crystal. This implies $J^{\mathrm{BB}}(\mathbf{k}) = m_z\, J^{\mathrm{AA}}(\mathbf{k})\, m_z = J^{\mathrm{AA}}(k_x, k_y, -k_z)$. One can also show that $J^{\mathrm{BA}}(\mathbf{k}) = J^{\mathrm{AB}}(\mathbf{k})$ using $J^{\mathrm{BA}}_{0j} = J^{\mathrm{AB}}_{j0} = J^{\mathrm{AB}}_{0-j} = J^{\mathrm{AB}}_{0j}$.

The dominant exchange interactions according to the DFT simulations are listed in Table SI. The vector connecting the two magnetic sites is given by $\mathbf{R}^{\mu\nu}_{0j} = x\,\mathbf{a} + y\,\mathbf{b} + z\,\mathbf{c}$ in terms of the cell vectors defining the hexagonal unit cell. The interaction shells which are

label	(x, y, z)	d [Å]	n	J [meV]
J_1^{AA}	$(1, 0, 0)$	4.1	6	9.2
J_2^{AA}	$(0, 0, 1)$	5.5	2	−3.5
J_3^{AA}	$(1, 0, 1)$	6.8	12	0.7
J_4^{AA}	$(2, 1, 0)$	7.1	6	−0.8
J_5^{AA}	$(2, 0, 0)$	8.2	6	0.2
J_6^{AA}	$(2, 1, 1)$	9.0	6	−0.1
J_7^{AA}	$(2, 1, -1)$	9.0	6	−2.7
J_1^{AB}	$(0, 0, {}^1\!/_2)$	2.7	2	−19.0
J_2^{AB}	$(1, 0, {}^1\!/_2)$	4.9	12	−5.3
J_3^{AB}	$(2, 1, {}^1\!/_2)$	7.6	12	0.9
J_4^{AB}	$(0, 0, {}^3\!/_2)$	8.2	2	−0.1
J_5^{AB}	$(2, 0, {}^1\!/_2)$	8.6	12	0.2
J_6^{AB}	$(1, 0, {}^3\!/_2)$	9.2	12	0.3

Table SI. **Computed exchange interactions.** The columns list the label used for the coupling, fractional coordinates for a representative bond vector, the distance d between the magnetic sites, how many symmetry-equivalent pairs are present (n), and the value of the respective exchange interaction J according to the DFT simulations. Positive and negative values indicate ferromagnetic and antiferromagnetic coupling, respectively.

not invariant under m_z are $J_6^{\rm AA}$ and $J_7^{\rm AA}$, and $J_6^{\rm BB} = m_z\, J_7^{\rm AA}\, m_z$. The difference between $J_6^{\rm AA}$ and $J_7^{\rm AA}$ is responsible for the altermagnetic splitting of the magnon spectrum.

C. Altermagnetic magnons

The following derivation is adapted from the book of Nolting and Ramakanth [9] to the symmetry of CrSb. Applying the Holstein-Primakoff transformation up to quadratic order to the spins on each sublattice,

$$S_{iz}^{\rm A} = +S - a_i^\dagger a_i \ , \quad S_{i+}^{\rm A} = \sqrt{2S}\, a_i \ , \tag{S4}$$

$$S_{iz}^{\rm B} = -S + b_i^\dagger b_i \ , \quad S_{i+}^{\rm B} = \sqrt{2S}\, b_i^\dagger \ . \tag{S5}$$

to the spin Hamiltonian

$$\mathcal{H} = -\frac{1}{NS^2} \sum_{i,j} \sum_{\mu,\nu} J_{ij}^{\mu\nu} \left(S_{iz}^{\mu} S_{jz}^{\nu} + \frac{S_{i+}^{\mu} S_{j-}^{\nu} + S_{i-}^{\mu} S_{j+}^{\nu}}{2} \right) \ . \tag{S6}$$

we get, using $J_{ij}^{\mu\nu} = J_{ji}^{\nu\mu}$, the commutation relations and some relabeling,

$$\begin{aligned} \mathcal{H} = &-\frac{1}{N} \sum_{i,j} \left(J_{ij}^{\rm AA} + J_{ij}^{\rm BB} - J_{ij}^{\rm AB} - J_{ij}^{\rm BA} \right) \\ &+ \frac{2}{NS} \sum_{i,j} \left((J_{ij}^{\rm AA} - J_{ij}^{\rm AB})\, a_i^\dagger a_i + (J_{ij}^{\rm BB} - J_{ij}^{\rm BA})\, b_i^\dagger b_i \right) \\ &- \frac{2}{NS} \sum_{i,j} \left(J_{ij}^{\rm AA}\, a_i^\dagger a_j + J_{ij}^{\rm BB}\, b_i^\dagger b_j + J_{ij}^{\rm AB} (a_i b_j + a_i^\dagger b_j^\dagger) \right) \ . \end{aligned} \tag{S7}$$

Rewriting using the lattice Fourier transform:

$$a_i = \sum_{\mathbf{k}} e^{\mathrm{i}\mathbf{k}\cdot(\mathbf{R}_i + \mathbf{R}_{\rm A})} a_{\mathbf{k}} \ , \quad b_i = \sum_{\mathbf{k}} e^{-\mathrm{i}\mathbf{k}\cdot(\mathbf{R}_i + \mathbf{R}_{\rm B})} b_{\mathbf{k}} \ , \tag{S8}$$

leads to, using $J^{\mu\nu}(-\mathbf{k}) = J^{\mu\nu}(\mathbf{k})$,

$$\mathcal{H} = E_0 + \frac{2}{S} \sum_{\mathbf{k}} \left(\left(B^{\rm eff} - J^{\rm AA}(\mathbf{k}) \right) a_{\mathbf{k}}^\dagger a_{\mathbf{k}} + \left(B^{\rm eff} - J^{\rm BB}(\mathbf{k}) \right) b_{\mathbf{k}}^\dagger b_{\mathbf{k}} - J^{\rm AB}(\mathbf{k}) \left(a_{\mathbf{k}} b_{\mathbf{k}} + a_{\mathbf{k}}^\dagger b_{\mathbf{k}}^\dagger \right) \right) \ . \tag{S9}$$

with $B^{\rm eff} = J^{\rm AA}(0) - J^{\rm AB}(0)$ and $E_0 = -2B^{\rm eff}$.

The Hamiltonian has the structure

$$\mathcal{H} = E_0 + \sum_{\mathbf{k}} \left(A(\mathbf{k})\, a_{\mathbf{k}}^\dagger a_{\mathbf{k}} + B(\mathbf{k})\, b_{\mathbf{k}}^\dagger b_{\mathbf{k}} + C(\mathbf{k}) \left(a_{\mathbf{k}} b_{\mathbf{k}} + a_{\mathbf{k}}^\dagger b_{\mathbf{k}}^\dagger \right) \right) \ , \tag{S10}$$

and is diagonalized by a suitable Bogoliubov transformation,

$$\alpha_{\mathbf{k}} = u_{\mathbf{k}} a_{\mathbf{k}} + v_{\mathbf{k}} b^\dagger_{\mathbf{k}} \,, \quad \beta_{\mathbf{k}} = u_{\mathbf{k}} b_{\mathbf{k}} + v_{\mathbf{k}} a^\dagger_{\mathbf{k}} \,, \tag{S11}$$

leading to

$$\mathcal{H} = \widetilde{E}_0 + \sum_{\mathbf{k}} \left(\omega_+(\mathbf{k})\, \alpha^\dagger_{\mathbf{k}} \alpha_{\mathbf{k}} + \omega_-(\mathbf{k})\, \beta^\dagger_{\mathbf{k}} \beta_{\mathbf{k}} \right) \,. \tag{S12}$$

The transformation must preserve the commutation relations, which imposes $u_{\mathbf{k}}^2 - v_{\mathbf{k}}^2 = 1$. $\widetilde{E}_0$ is the ground state energy including the zero-point energy correction.

The coefficients can be found from

$$\begin{aligned} [\alpha_{\mathbf{k}}, \mathcal{H}] &= \omega_+(\mathbf{k})\, \alpha_{\mathbf{k}} && \text{(S13)} \\ &= \omega_+(\mathbf{k}) \left(u_{\mathbf{k}} a_{\mathbf{k}} + v_{\mathbf{k}} b^\dagger_{\mathbf{k}} \right) && \text{(S14)} \\ &= [u_{\mathbf{k}} a_{\mathbf{k}} + v_{\mathbf{k}} b^\dagger_{\mathbf{k}}, \mathcal{H}] && \text{(S15)} \\ &= u_{\mathbf{k}} \left(A(\mathbf{k})\, a_{\mathbf{k}} + C(\mathbf{k})\, b^\dagger_{\mathbf{k}} \right) - v_{\mathbf{k}} \left(B(\mathbf{k})\, b^\dagger_{\mathbf{k}} + C(\mathbf{k}) a_{\mathbf{k}} \right) \,, && \text{(S16)} \end{aligned}$$

and setting the coefficients in front of the $a_{\mathbf{k}}$ and $b^\dagger_{\mathbf{k}}$ operators to zero leads to the system of equations

$$\begin{pmatrix} \omega_+(\mathbf{k}) - A(\mathbf{k}) & C(\mathbf{k}) \\ -C(\mathbf{k}) & \omega_+(\mathbf{k}) + B(\mathbf{k}) \end{pmatrix} \begin{pmatrix} u_{\mathbf{k}} \\ v_{\mathbf{k}} \end{pmatrix} = 0 \,, \tag{S17}$$

which has a solution when

$$\omega_+(\mathbf{k}) = \sqrt{\left(\frac{A(\mathbf{k}) + B(\mathbf{k})}{2} \right)^2 - C(\mathbf{k})^2} + \frac{A(\mathbf{k}) - B(\mathbf{k})}{2} = \omega^{\mathrm{AF}}(\mathbf{k}) + \Delta(\mathbf{k}) \,, \tag{S18}$$

leading to

$$u_{\mathbf{k}} = \frac{\omega_+(\mathbf{k}) + B(\mathbf{k})}{\sqrt{(\omega_+(\mathbf{k}) + B(\mathbf{k}))^2 - C(\mathbf{k})^2}} \,, \quad v_{\mathbf{k}} = \frac{C(\mathbf{k})}{\sqrt{(\omega_+(\mathbf{k}) + B(\mathbf{k}))^2 - C(\mathbf{k})^2}} \,, \tag{S19}$$

which satisfy $|u_{\mathbf{k}}|^2 - |v_{\mathbf{k}}|^2 = 1$. Following the same steps for $\beta_{\mathbf{k}}$ leads to

$$\omega_-(\mathbf{k}) = \omega^{\mathrm{AF}}(\mathbf{k}) - \Delta(\mathbf{k}) \,, \tag{S20}$$

with the same $u_{\mathbf{k}}$ and $v_{\mathbf{k}}$ as before. Substituting back the definitions,

$$\omega^{\mathrm{AF}}(\mathbf{k}) = \frac{2}{S} \sqrt{\left(B^{\mathrm{eff}} - \frac{J^{\mathrm{AA}}(\mathbf{k}) + J^{\mathrm{BB}}(\mathbf{k})}{2} \right)^2 - J^{\mathrm{AB}}(\mathbf{k})^2} \,, \tag{S21}$$

$$\Delta(\mathbf{k}) = \frac{1}{S} \left(J^{\mathrm{BB}}(\mathbf{k}) - J^{\mathrm{AA}}(\mathbf{k}) \right) \,. \tag{S22}$$

This analysis shows that the difference between the conventional antiferromagnetic magnons and the altermagnetic magnons is the existence of $\Delta(\mathbf{k}) \propto J^{\mathrm{BB}}(\mathbf{k}) - J^{\mathrm{AA}}(\mathbf{k})$.

To express sublattice properties in terms of the new magnon operators we have to invert the Bogoliubov transformation:

$$\begin{pmatrix} \alpha_{\mathbf{k}} \\ \beta^{\dagger}_{\mathbf{k}} \end{pmatrix} = \begin{pmatrix} u_{\mathbf{k}} & v_{\mathbf{k}} \\ v_{\mathbf{k}} & u_{\mathbf{k}} \end{pmatrix} \begin{pmatrix} a_{\mathbf{k}} \\ b^{\dagger}_{\mathbf{k}} \end{pmatrix} \longrightarrow \begin{pmatrix} a_{\mathbf{k}} \\ b^{\dagger}_{\mathbf{k}} \end{pmatrix} = \begin{pmatrix} u_{\mathbf{k}} & -v_{\mathbf{k}} \\ -v_{\mathbf{k}} & u_{\mathbf{k}} \end{pmatrix} \begin{pmatrix} \alpha_{\mathbf{k}} \\ \beta^{\dagger}_{\mathbf{k}} \end{pmatrix} . \tag{S23}$$

The spin deviation on each sublattice caused by a given magnon can be obtained from:

$$a^{\dagger}_{\mathbf{k}} a_{\mathbf{k}} = v^2_{\mathbf{k}} + u^2_{\mathbf{k}}\, \alpha^{\dagger}_{\mathbf{k}} \alpha_{\mathbf{k}} - v^2_{\mathbf{k}}\, \beta^{\dagger}_{\mathbf{k}} \beta_{\mathbf{k}} - u_{\mathbf{k}} v_{\mathbf{k}} \left(\alpha_{\mathbf{k}} \beta_{\mathbf{k}} + \alpha^{\dagger}_{\mathbf{k}} \beta^{\dagger}_{\mathbf{k}} \right) , \tag{S24}$$

$$b^{\dagger}_{\mathbf{k}} b_{\mathbf{k}} = v^2_{\mathbf{k}} + u^2_{\mathbf{k}}\, \beta^{\dagger}_{\mathbf{k}} \beta_{\mathbf{k}} - v^2_{\mathbf{k}}\, \alpha^{\dagger}_{\mathbf{k}} \alpha_{\mathbf{k}} - u_{\mathbf{k}} v_{\mathbf{k}} \left(\alpha_{\mathbf{k}} \beta_{\mathbf{k}} + \alpha^{\dagger}_{\mathbf{k}} \beta^{\dagger}_{\mathbf{k}} \right) , \tag{S25}$$

by inserting back into Eq. S4. The constant $v^2_{\mathbf{k}}$ is the quantum correction to the classical Néel state assumed to have local spin projection $S_z = \pm S$. If $u^2_{\mathbf{k}} > v^2_{\mathbf{k}}$ this shows that the α magnon has a larger amplitude on sublattice A than on sublattice B, and conversely for the β magnon. The anomalous terms make no contribution for expectation values involving the same magnon states.

D. RIXS and dichroism of the magnon peak

Using the Kramers-Heisenberg formula and the dipole and rotating wave approximations, the direct RIXS cross section can be written as [10]

$$\frac{\mathrm{d}^2\sigma}{\mathrm{d}\Omega_f \mathrm{d}\omega_f} \propto \sum_f \left| \sum_e \frac{\langle f | \mathcal{H}^{-}_{\mathrm{em},f} | e \rangle \langle e | \mathcal{H}^{+}_{\mathrm{em},i} | i \rangle}{E_i + \omega_i - E_e + \mathrm{i}\Gamma_e} \right|^2 \delta(\omega_f + E_f - E_i - \omega_i) . \tag{S26}$$

The interaction between x-rays and matter is given by $\mathcal{H}_{\mathrm{em}}$. The incident photon has frequency ω_i and the electronic system is in the ground state $|i\rangle$ with energy E_i. Once the photon is absorbed ($\mathcal{H}^{+}_{\mathrm{em},i}$) the electronic system jumps to an excited state $|e\rangle$ with energy E_e and lifetime Γ_e, before de-exciting to a final state $|f\rangle$ with energy E_f by emitting a photon ($\mathcal{H}^{-}_{\mathrm{em},f}$) with frequency ω_f into the solid angle Ω_f. All possible final states are considered by the summation. Energy conservation is enforced by the delta function; this means that $\omega_f < \omega_i$ as $E_f > E_i$, being an excited state.

The connection between RIXS and the dynamical spin correlation function has been discussed by several authors, and here we adapt the arguments by Haverkort [11]. The idea

is to introduce an effective low-energy scattering operator

$$\sum_e \frac{\langle f|\mathcal{H}^-_{\mathrm{em},f}|e\rangle\,\langle e|\mathcal{H}^+_{\mathrm{em},i}|i\rangle}{E_i+\omega_i-E_e+\mathrm{i}\Gamma_e} \longrightarrow \langle f|\mathcal{R}|i\rangle \tag{S27}$$

that replaces the explicit sum over excited states by operators that connect directly the initial and final states. Letting $\boldsymbol{\epsilon}_i$ and $\boldsymbol{\epsilon}_f$ be the polarizations of the incoming and outgoing photons and $\mathbf{k}_i$ and $\mathbf{k}_f$ their wave vectors, respectively, the effective scattering operator for a given atom in sublattice μ has the form $\mathcal{R}^\mu = \mathcal{R}^\mu_0 + \mathcal{R}^\mu_{\mathrm{m}}$, with $\mathcal{R}^\mu_0$ describing charge scattering and the magnetic scattering being given by

$$\mathcal{R}^\mu_{\mathrm{m}} \propto F_{a_{2u}}(\boldsymbol{\epsilon}^*_f\times\boldsymbol{\epsilon}_i)_z S^\mu_{jz} + F_{e_u}\left((\boldsymbol{\epsilon}^*_f\times\boldsymbol{\epsilon}_i)_+ S^\mu_{j-} + (\boldsymbol{\epsilon}^*_f\times\boldsymbol{\epsilon}_i)_- S^\mu_{j+}\right) + \mathcal{O}(S^2)\,. \tag{S28}$$

Here $F_{a_{2u}}$ and F_{e_u} describe the two independent fundamental X-ray dichroic spectra allowed by the $\bar{3}m$ symmetry of the environment of each Cr atom, which are sublattice independent. The total effective scattering operator is then obtained from

$$\frac{1}{N}\sum_j\sum_\mu e^{\mathrm{i}\mathbf{q}\cdot(\mathbf{R}_j+\mathbf{R}_\mu)}\mathcal{R}^\mu_j = \sum_\mu \mathcal{R}^\mu_{\mathbf{q}}\,, \tag{S29}$$

with $\mathbf{q}=\mathbf{k}_i-\mathbf{k}_f$. The spin dependence is given by

$$\mathcal{R}^\mu_{\mathrm{m},\mathbf{q}} = \frac{1}{N}\sum_j e^{\mathrm{i}\mathbf{q}\cdot(\mathbf{R}_j+\mathbf{R}_\mu)}\left(\mathcal{R}^{fi}_z S^\mu_{jz} + \mathcal{R}^{fi}_+ S^\mu_{j-} + \mathcal{R}^{fi}_- S^\mu_{j+}\right)\,. \tag{S30}$$

For each sublattice we then find

$$\mathcal{R}^{\mathrm{A}}_{\mathrm{m},\mathbf{q}} = \mathcal{R}^{fi}_z\left(S\,\delta_{\mathbf{q},\mathbf{0}} - \sum_{\mathbf{k}} a^\dagger_{\mathbf{k}+\mathbf{q}}a_{\mathbf{k}}\right) + \sqrt{2S}\left(\mathcal{R}^{fi}_+ a^\dagger_{\mathbf{q}} + \mathcal{R}^{fi}_- a_{-\mathbf{q}}\right)\,, \tag{S31}$$

$$\mathcal{R}^{\mathrm{B}}_{\mathrm{m},\mathbf{q}} = \mathcal{R}^{fi}_z\left(-S\,\delta_{\mathbf{q},\mathbf{0}} + \sum_{\mathbf{k}} b^\dagger_{\mathbf{k}-\mathbf{q}}b_{\mathbf{k}}\right) + \sqrt{2S}\left(\mathcal{R}^{fi}_+ b_{\mathbf{q}} + \mathcal{R}^{fi}_- b^\dagger_{-\mathbf{q}}\right)\,. \tag{S32}$$

To excite a single magnon we keep only terms linear in the Holstein-Primakoff bosons. To proceed we substitute for the new operators obtained by the Bogoliubov transformation:

$$\mathcal{R}^{\mathrm{A}}_{\mathrm{m},\mathbf{q}} \approx \sqrt{2S}\,\mathcal{R}^{fi}_+\left(u_{\mathbf{q}}\alpha^\dagger_{\mathbf{q}} - v_{\mathbf{q}}\beta_{\mathbf{q}}\right) + \sqrt{2S}\,\mathcal{R}^{fi}_-\left(u_{-\mathbf{q}}\alpha_{-\mathbf{q}} - v_{-\mathbf{q}}\beta^\dagger_{-\mathbf{q}}\right)\,, \tag{S33}$$

$$\mathcal{R}^{\mathrm{B}}_{\mathrm{m},\mathbf{q}} \approx \sqrt{2S}\,\mathcal{R}^{fi}_+\left(u_{\mathbf{q}}\beta_{\mathbf{q}} - v_{\mathbf{q}}\alpha^\dagger_{\mathbf{q}}\right) + \sqrt{2S}\,\mathcal{R}^{fi}_-\left(u_{-\mathbf{q}}\beta^\dagger_{-\mathbf{q}} - v_{-\mathbf{q}}\alpha_{-\mathbf{q}}\right)\,. \tag{S34}$$

Inserting this into the RIXS cross section gives ($\omega=\omega_i-\omega_f>0$ for energy absorption)

$$\begin{aligned}\frac{\mathrm{d}^2\sigma}{\mathrm{d}\Omega\mathrm{d}\omega} &\propto \sum_{\mu,\nu}\sum_f \left(\langle f|\mathcal{R}^\mu_{\mathrm{m},\mathbf{q}}|i\rangle\right)^*\langle f|\mathcal{R}^\nu_{\mathrm{m},\mathbf{q}}|i\rangle\,\delta(E_f-E_i-\omega)\\ &= 2S\left(u^2_{\mathbf{q}}+v^2_{\mathbf{q}}-2u_{\mathbf{q}}v_{\mathbf{q}}\right)\sum_f\sum_{s=\pm}\left|\mathcal{R}^{fi}_s\right|^2(1+n_{\mathbf{q}s})\,\delta(\omega-\omega_{\mathbf{q}s})\,,\end{aligned} \tag{S35}$$

where inversion symmetry was used to convert $-\mathbf{q}$ to $+\mathbf{q}$ where needed. Here $n_{\mathbf{q}s}$ are thermal occupation factors given by the Bose-Einstein distribution. The remaining f-summation is over the polarization states of the outgoing photon.

The polarization factors are worked out as follows. Expressing the propagation direction of the incoming and outgoing photons as

$$\hat{\mathbf{k}}_i = \cos\theta_i\,(\cos\phi_i\,\hat{\mathbf{x}} + \sin\phi_i\,\hat{\mathbf{y}}) + \sin\theta_i\,\hat{\mathbf{z}}\ , \tag{S36}$$

$$\hat{\mathbf{k}}_f = \cos\theta_f\,(\cos\phi_f\,\hat{\mathbf{x}} + \sin\phi_f\,\hat{\mathbf{y}}) - \sin\theta_f\,\hat{\mathbf{z}}\ , \tag{S37}$$

the two independent linear polarizations can be expressed in terms of the photon wave vector and the normal to the surface, assumed to be $+\hat{\mathbf{z}}$ as in Fig. 1c of the main text:

$$\hat{\boldsymbol{\sigma}} = \frac{\hat{\mathbf{z}}\times\hat{\mathbf{k}}}{\cos\theta}\ ,\quad \hat{\boldsymbol{\pi}} = \hat{\mathbf{k}}\times\hat{\boldsymbol{\sigma}}\ ,\quad \hat{\boldsymbol{\sigma}}\times\hat{\boldsymbol{\pi}} = \hat{\mathbf{k}}\ . \tag{S38}$$

For the incoming photon this gives

$$\hat{\boldsymbol{\sigma}}_i = -\sin\phi_i\,\hat{\mathbf{x}} + \cos\phi_i\,\hat{\mathbf{y}}\ ,\quad \hat{\boldsymbol{\pi}}_i = -\sin\theta_i\,(\cos\phi_i\,\hat{\mathbf{x}} + \sin\phi_i\,\hat{\mathbf{y}}) + \cos\theta_i\,\hat{\mathbf{z}}\ , \tag{S39}$$

$$\boldsymbol{\epsilon}_p = \hat{\boldsymbol{\sigma}}_i + \mathrm{i}p\,\hat{\boldsymbol{\pi}}_i\ , \tag{S40}$$

where the circular polarizations have been introduced with $p = \pm$. For the outgoing photon we have

$$\hat{\boldsymbol{\sigma}}_f = -\sin\phi_f\,\hat{\mathbf{x}} + \cos\phi_f\,\hat{\mathbf{y}}\ ,\quad \hat{\boldsymbol{\pi}}_f = +\sin\theta_f\,(\cos\phi_f\,\hat{\mathbf{x}} + \sin\phi_f\,\hat{\mathbf{y}}) + \cos\theta_f\,\hat{\mathbf{z}}\ . \tag{S41}$$

Then we can consider separately the two polarizations of the outgoing photon, and add up the results at the end.

For an outgoing photon with σ polarization, $\boldsymbol{\epsilon}_f^* \times \boldsymbol{\epsilon}_i = \hat{\boldsymbol{\sigma}}_f \times \boldsymbol{\epsilon}_p$. The various products of polarization vectors give

$$\hat{\boldsymbol{\sigma}}_f \times \hat{\boldsymbol{\sigma}}_i = \sin\phi_{if}\,\hat{\mathbf{z}}\ , \tag{S42}$$

$$\hat{\boldsymbol{\sigma}}_f \times \hat{\boldsymbol{\pi}}_i = \cos\theta_i\,(\cos\phi_f\,\hat{\mathbf{x}} + \sin\phi_f\,\hat{\mathbf{y}}) + \sin\theta_i\cos\phi_{fi}\,\hat{\mathbf{z}}\ , \tag{S43}$$

with $\phi_{fi} = \phi_f - \phi_i$, and so ($p^2 = 1$)

$$\big(\boldsymbol{\epsilon}_f^* \times \boldsymbol{\epsilon}_i\big)_\pm = (\hat{\boldsymbol{\sigma}}_f \times \boldsymbol{\epsilon}_p)\cdot(\hat{\mathbf{x}} + \mathrm{i}s\,\hat{\mathbf{y}}) = \mathrm{i}p\cos\theta_i\,e^{\mathrm{i}s\phi_f} \quad\longrightarrow\quad \big|\mathcal{R}^{fi}_{\sigma p,s}\big|^2 \propto \cos^2\theta_i\ . \tag{S44}$$

For an outgoing photon with π polarization,

$$\hat{\boldsymbol{\pi}}_f \times \hat{\boldsymbol{\sigma}}_i = -\cos\theta_f \left(\cos\phi_i\,\hat{\mathbf{x}} + \sin\phi_i\,\hat{\mathbf{y}}\right) + \sin\theta_f \cos\phi_{fi}\,\hat{\mathbf{z}}\ , \tag{S45}$$

$$\begin{aligned}\hat{\boldsymbol{\pi}}_f \times \hat{\boldsymbol{\pi}}_i &= \sin\theta_f \cos\theta_i \left(\sin\phi_f\,\hat{\mathbf{x}} - \cos\phi_f\,\hat{\mathbf{y}}\right) + \cos\theta_f \sin\theta_i \left(\sin\phi_i\,\hat{\mathbf{x}} - \cos\phi_i\,\hat{\mathbf{y}}\right) \\ &\quad + \sin\theta_f \sin\theta_i \sin\phi_{fi}\,\hat{\mathbf{z}}\ .\end{aligned} \tag{S46}$$

The piece of interest is

$$(\hat{\boldsymbol{\pi}}_f \times \boldsymbol{\epsilon}_p)\cdot(\hat{\mathbf{x}} + \mathrm{i}s\,\hat{\mathbf{y}}) = -\cos\theta_f\, e^{\mathrm{i}s\phi_f} + sp\left(\sin\theta_f \cos\theta_i\, e^{\mathrm{i}s\phi_f} + \cos\theta_f \sin\theta_i\, e^{\mathrm{i}s\phi_i}\right)\ , \tag{S47}$$

and so

$$\begin{aligned}\left|\mathcal{R}^{fi}_{\pi p,s}\right|^2 &\propto \cos^2\theta_f + \cos^2\theta_f \sin^2\theta_i + \sin^2\theta_f \cos^2\theta_i - \frac{1}{2}\sin 2\theta_f \sin 2\theta_i \cos\phi_{fi} \\ &\quad - 2sp\left(\cos^2\theta_f \sin\theta_i + \cos\theta_f \sin\theta_f \cos\theta_i \cos\phi_{ij}\right)\ .\end{aligned} \tag{S48}$$

As the outgoing polarization is not detected, we sum both contributions

$$\begin{aligned}\sum_f \left|\mathcal{R}^{fi}_s\right|^2 &= \left|\mathcal{R}^{fi}_{\sigma p,s}\right|^2 + \left|\mathcal{R}^{fi}_{\pi p,s}\right|^2 \\ &\propto \frac{1}{2}\left(3 + \cos 2\theta_f + \cos 2\theta_i - \cos 2\theta_f \cos 2\theta_i - \sin 2\theta_f \sin 2\theta_i \cos\phi_{fi}\right) \\ &\quad - sp\left((1+\cos 2\theta_f)\sin\theta_i + \sin 2\theta_f \cos\theta_i \cos\phi_{ij}\right) \qquad \text{(S49)} \\ &= f_0(\hat{\mathbf{k}}_i, \hat{\mathbf{k}}_f) - sp\, f_1(\hat{\mathbf{k}}_i, \hat{\mathbf{k}}_f)\ . \qquad \text{(S50)}\end{aligned}$$

The coupling between the circular polarization of the photons (p) and the circular polarization of the magnons (s) is given by the last term, and so is responsible for the dichroism.

We can finally return to the RIXS cross section related to the magnons:

$$\frac{\mathrm{d}^2\sigma}{\mathrm{d}\Omega \mathrm{d}\omega} \propto A(\mathbf{q})\left[f_0(\hat{\mathbf{k}}_i, \hat{\mathbf{k}}_f)\big(L_-(\mathbf{q},\omega) + L_+(\mathbf{q},\omega)\big) + p\, f_1(\hat{\mathbf{k}}_i, \hat{\mathbf{k}}_f)\big(L_-(\mathbf{q},\omega) - L_+(\mathbf{q},\omega)\big)\right]\ , \tag{S51}$$

with the prefactor $A(\mathbf{q}) = 2S\left(u_{\mathbf{q}}^2 + v_{\mathbf{q}}^2 - 2\,u_{\mathbf{q}} v_{\mathbf{q}}\right)$ and the spectral functions

$$L_s(\mathbf{q},\omega) = (1 + n_{\mathbf{q}s})\,\delta(\omega - \omega_{\mathbf{q}s}) \longrightarrow (1 + n_{\mathbf{q}s})\,\frac{\omega\Gamma_{\mathbf{q}s}/\pi}{(\omega - \omega_{\mathbf{q}s})^2 + \Gamma_{\mathbf{q}s}^2}\ , \tag{S52}$$

accounting for broadening with a skewed Lorentzian of width $\Gamma_{\mathbf{q}s}$.

For the relative circular dichroism this gives

$$R_{\mathrm{CD}}(\mathbf{q},\omega) = \frac{f_1(\hat{\mathbf{k}}_i, \hat{\mathbf{k}}_f)\big(L_-(\mathbf{q},\omega) - L_+(\mathbf{q},\omega)\big)}{f_0(\hat{\mathbf{k}}_i, \hat{\mathbf{k}}_f)\big(L_-(\mathbf{q},\omega) + L_+(\mathbf{q},\omega)\big)}\ . \tag{S53}$$

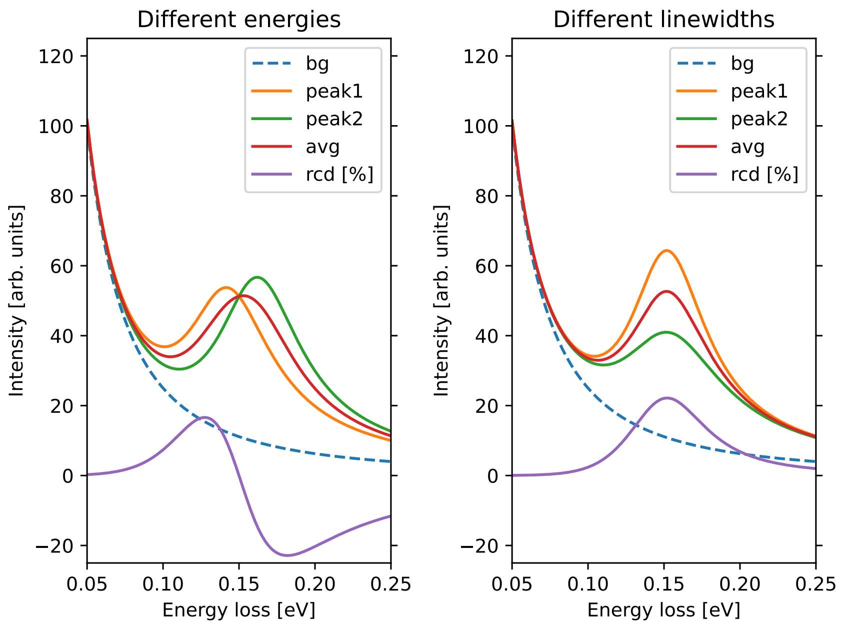

Fig. S4. Two possible scenarios for circular dichroism in the RIXS spectra. (a) Peaks centered at different energies but with same broadening: $\omega_0 = 0.14, 0.16$ eV, $\Gamma = 0.033$ eV. (b) Peaks centered at same energy but with different broadenings: $\omega_0 = 0.15$ eV, $\Gamma = 0.03, 0.04$ eV. The peaks are given by $I_{\mathrm{peak}}(\omega) = \frac{\omega/\pi}{(\omega-\omega_0)^2+\Gamma^2}$. In both scenarios a simple background representing the tail of the elastic line was included, with the form $I_{\mathrm{bg}}(\omega) = \frac{0.5}{\omega^2}$. The peak average and the relative difference, Eq. (S53), are also shown.

For our azimuthal scans the factors $f_0(\hat{\mathbf{k}}_i, \hat{\mathbf{k}}_f)$ and $f_1(\hat{\mathbf{k}}_i, \hat{\mathbf{k}}_f)$ are constant, and the ϕ-dependence of the dichroism can only originate from the q-dependence of the spectral functions. The background to the magnon signal should be added in the denominator, for comparison with experimental results. A simple toy model for the expected behaviour of $R_{\mathrm{CD}}(\mathbf{q}, \omega)$ is presented in Fig. S4, which shows the different shape of the dichroism that results from an asymmetry due to a large magnon energy splitting, Fig. S4(a), and from a magnon linewidth asymmetry, Fig. S4(b).

SUPPLEMENTARY REFERENCES

[1] K.-J. Zhou, A. Walters, M. Garcia-Fernández, T. Rice, M. Hand, A. Nag, J. Li, S. Agrestini, P. Garland, H. Wang, S. Alcock, I. Nistea, B. Nutter, N. Rubies, G. Knap, M. Gaughran,

F. Yuan, P. Chang, J. Emmins, and G. Howell, I21: an advanced high-resolution resonant inelastic X-ray scattering beamline at Diamond Light Source, J. Synchrotron Radiat. **29**, 563 (2022).

[2] T. Hayashida, K. Arakawa, T. Oshima, K. Kimura, and T. Kimura, Observation of antiferromagnetic domains in Cr_2O_3 using nonreciprocal optical effects, Phys. Rev. Res. **4**, 043063 (2022).

[3] https://jukkr.fz-juelich.de.

[4] N. Papanikolaou, R. Zeller, and P. H. Dederichs, Conceptual improvements of the KKR method, J. Phys.: Condens. Matter **14**, 2799 (2002).

[5] D. S. G. Bauer, *Development of a relativistic full-potential first-principles multiple scattering Green function method applied to complex magnetic textures of nano structures at surfaces*, Ph.D. thesis, RWTH Aachen (2014).

[6] S. H. Vosko, L. Wilk, and M. Nusair, Accurate spin-dependent electron liquid correlation energies for local spin density calculations: a critical analysis, Can. J. Phys. **58**, 1200 (1980).

[7] K. Wildberger, P. Lang, R. Zeller, and P. H. Dederichs, Fermi-Dirac distribution in ab initio Green's-function calculations, Phys. Rev. B **52**, 11502 (1995).

[8] H. Ebert and S. Mankovsky, Anisotropic exchange coupling in diluted magnetic semiconductors: Ab initio spin-density functional theory, Phys. Rev. B **79**, 045209 (2009).

[9] W. Nolting and A. Ramakanth, *Quantum Theory of Magnetism* (Springer Berlin Heidelberg, 2009).

[10] F. M. F. de Groot, M. W. Haverkort, H. Elnaggar, A. Juhin, K.-J. Zhou, and P. Glatzel, Resonant inelastic X-ray scattering, Nat. Rev. Methods Primers **4**, 45 (2024).

[11] M. W. Haverkort, Theory of Resonant Inelastic X-Ray Scattering by Collective Magnetic Excitations, Phys. Rev. Lett. **105**, 167404 (2010).
